# X-Linked RNA-Binding Motif Protein Modulates HIV-1 Infection of CD4^+^ T Cells by Maintaining the Trimethylation of Histone H3 Lysine 9 at the Downstream Region of the 5′ Long Terminal Repeat of HIV Proviral DNA

**DOI:** 10.1128/mBio.03424-19

**Published:** 2020-04-21

**Authors:** Li Ma, Qing-An Jiang, Li Sun, Xianguang Yang, Hai Huang, Xia Jin, Chiyu Zhang, Jian-Hua Wang

**Affiliations:** a School of Life Science, Shanghai University, Shanghai, China; b CAS Key Laboratory of Molecular Virology and Immunology, Institut Pasteur of Shanghai, Chinese Academy of Sciences, Shanghai, China; c College of Life Science, Henan Normal University, Xinxiang, Henan Province, China; d University of Chinese Academy of Sciences, Beijing, China; McMaster University

**Keywords:** HIV-1, RBMX, transcription, H3K9me3, HIV-1

## Abstract

Reversible repression of HIV-1 5′ long terminal repeat (5′-LTR)-mediated transcription represents the main mechanism for HIV-1 to maintain latency. Identification of host factors that modulate LTR activity and viral latency may help develop new antiretroviral therapies. The heterogeneous nuclear ribonucleoproteins (hnRNPs) are known to regulate gene expression and possess multiple physiological functions. hnRNP family members have recently been identified as the sensors for viral nucleic acids to induce antiviral responses, highlighting the crucial roles of hnRNPs in regulating viral infection. A member of the hnRNP family, X-linked RNA-binding motif protein (RBMX), has been identified in this study as a novel HIV-1 restriction factor that modulates HIV-1 5′-LTR-driven transcription of viral genome in CD4^+^ T cells. Mechanistically, RBMX binds to HIV-1 proviral DNA at the LTR downstream region and maintains the repressive trimethylation of histone H3 lysine 9 (H3K9me3), leading to a blockage of the recruitment of the positive transcription factor phosphorylated RNA polymerase II (RNA pol II) and consequential impediment of transcription elongation. This RBMX-mediated modulation of HIV-1 transcription maintains viral latency by inhibiting viral reactivation from an integrated proviral DNA. Our findings provide a new understanding of how host factors modulate HIV-1 infection and latency and suggest a potential new target for the development of HIV-1 therapies.

## Abstract (precis)

HIV-1 latency featuring silence of transcription from HIV-1 proviral DNA represents a major obstacle for HIV-1 eradication. Reversible repression of HIV-1 5′-LTR-mediated transcription represents the main mechanism for HIV-1 to maintain latency. The 5′-LTR-driven HIV gene transcription can be modulated by multiple host factors and mechanisms. The hnRNPs are known to regulate gene expression. A member of the hnRNP family, RBMX, has been identified in this study as a novel HIV-1 restriction factor that modulates HIV-1 5′-LTR-driven transcription of viral genome in CD4^+^ T cells and maintains viral latency. These findings provide a new understanding of how host factors modulate HIV-1 infection and latency and suggest a potential new target for the development of HIV-1 therapies.

## INTRODUCTION

HIV-1 latency featuring silence of transcription from HIV-1 proviral DNA represents a major obstacle for HIV-1 eradication (1–4). Eradicating latent HIV reservoir by activating viral gene transcription has recently been explored as a new adjunct to antiretroviral therapy. The 5′ long terminal repeat (5′-LTR)-driven HIV gene transcription can be modulated by multiple host factors and mechanisms. For example, epigenetic modification of the 5′-LTR of HIV-1 proviral DNA tightly regulates viral gene transcription (5, 6). This may be accomplished by recruitment of histone deacetylases, histone methyltransferases, or DNA methyltransferases; all of these enzymes can cause chromatin remodeling and nucleosome reorganization, leading to silencing of the viral promoter (6, 7). The heterochromatin-associated histone marker H3K9me3 (trimethylation of histone H3 lysine 9) is known to suppress HIV-1 transcription, and thus, a reduction of H3K9me3 modification by treatment with chaetocin, a lysine-specific histone methyltransferase, reactivates HIV from latency (8–10). Other chromatin reassembly factors, such as Spt6 (chromatin-specific transcription elongation factor SPT6), Chd1 (chromodomain-helicase-DNA-binding protein 1) and SUN2 (the Sad1 and UNC84 domain containing 2) have also been shown to modulate HIV-1 gene transcription by maintaining the heterochromatin (11–13).

Heterogeneous nuclear ribonucleoproteins (hnRNPs) are structurally and functionally distinct proteins containing specific domains and motifs that enable these proteins to bind certain nucleotide sequences and modulate gene expression, thereby modifying multiple physiological functions (14, 15). X-linked RNA-binding motif protein (RBMX) belongs to the hnRNP family of proteins (16, 17), and it was first identified as a glycosylated RNA-binding protein that is mainly located in the nucleus and nearly universally expressed in all tissues (18, 19). RBMX modulates multiple cellular processes. It has a highly conserved RNA-binding domain that enables its participation in pre-mRNA splicing (20). By associating with multiply splicing related proteins, RBMX forms the supraspliceosome that regulates the selection of alternative splice sites and facilities splicing of pre-mRNA (19, 21). Furthermore, RBMX has been reported to be an m6A reader protein that binds RNA through RRM and RGG (Arg-Gly-Gly) motifs (the low-complexity region), and it cotranscriptionally interacts with the phosphorylated RNA polymerase II (RNA pol II) and m6A-modifed nascent pre-mRNAs to modulate RNA pol II occupancy and alternative splicing (22, 23).

RBMX also participates in the regulation of gene transcription. In different cellular environments, RBMX may inhibit or activate gene transcription (20). For instance, RBMX is enriched within heterochromatin and interacts with transcriptionally modulated chromatin marker H3K9me3 to stop/slow down gene transcription during cellular reprogramming (24); conversely, through binding to the promoter of the sterol regulatory element-binding protein 1c (SREBP-1c) gene, RBMX activates transcription of the SREBP-1c gene in response to a high-fructose diet (25, 26).

By performing its functions in regulating transcription and pre-mRNA splicing, RBMX has been shown to perform differential activities in different viral infections. RBMX inhibits the infection of human papillomavirus type 16 by preventing the inclusion of exon on HPV16 late L1 mRNAs for splicing (27). In another setting, however, RBMX interacts with the nucleoprotein of Borna disease virus and promotes the formation of viral speckles of transcripts, leading to enhanced viral gene transcription (28). Which side of RBMX will affect HIV-1 infection is unknown.

In this study, we found that RBMX suppresses HIV-1 infection by modulating HIV-1 5′-LTR-driven viral transcription. At the molecular level, RBMX bound to HIV-1 proviral DNA at the downstream regions of LTR and maintained the repressive H3K9me3 modification. Consequently, the recruitment of the positive transcription factor was blocked and transcription elongation was stopped. At a functional level, RBMX-mediated modulation of HIV-1 transcription contributed to the maintenance of viral latency. Our findings facilitate a better understanding of host modulation of HIV-1 infection and latency and provide a potential host cell target for the development of HIV-1 therapies.

## RESULTS

### RBMX inhibits HIV-1 infection of CD4^+^ T cells.

**FIG 1:**
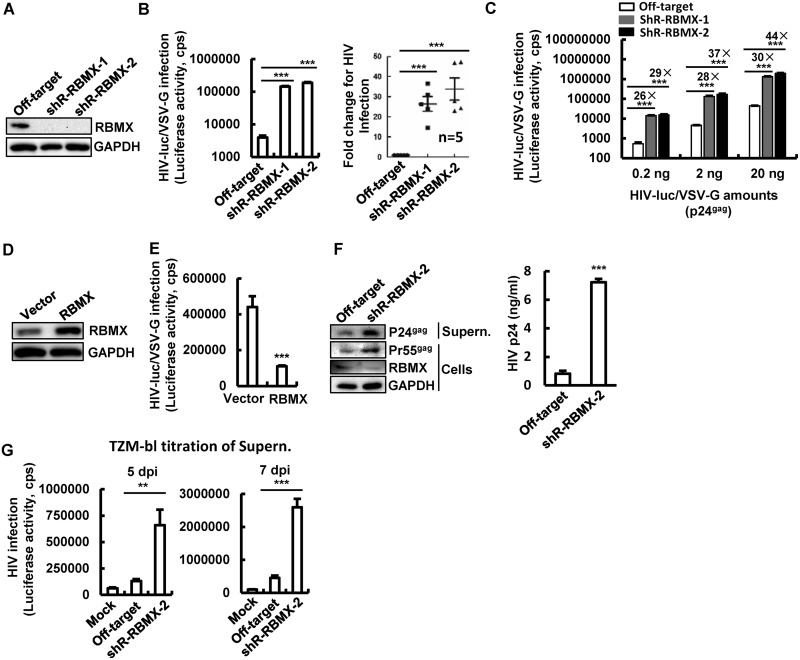
RBMX inhibits HIV-1 infection of CD4^+^ T cells. (A to C) RBMX knockdown promotes viral infection. Jurkat T cells were infected with lentiviruses containing RBMX-specific shRNAs, and RBMX expression was detected by Western blotting (A). Cells were infected with HIV-luc/VSV-G (2 ng p24^Gag^) for 48 h, viral replication was detected by measuring luciferase activity, and results from five independent experiments were summarized and analyzed (B). Cells were infected with the indicated amount of HIV-luc/VSV-G for 48 h (C). (D and E) RBMX overexpression suppresses HIV-1 infection. Jurkat T cells were transfected with RBMX-expressing plasmids by electroporation for 24 h and then infected with HIV-luc/VSV-G (2 ng p24^Gag^) for another 48 h. RBMX expression was detected by Western blotting (D), and HIV-1 infection was detected by measuring luciferase activity (E). (F and G) RBMX knockdown enhances HIV-1 replication in primary CD4^+^ T cells. PHA-P-activated primary CD4^+^ T cells were infected with lentiviruses containing RBMX-specific shRNAs for 3 days, then infected with replication-competent HIV_NL4-3_ (10 ng p24^Gag^) for an additional 5 or 7 days. Cell culture supernatants were harvested for detecting HIV-1 production by Western blotting or p24^gag^ capture ELISAs (F) or by titration in TZM-bl indicator cells (G). Western blotting was performed using samples from 7 days postinfection (F). Data are presented as mean ± standard deviations (SD) (error bars). The results from one experiment representative of at least three independent experiments is shown in panels C to G. Values that are significantly different are indicated by bars and asterisks as follows: **, *P* < 0.01; ***, *P* < 0.001. GAPDH, glyceraldehyde-3-phosphate dehydrogenase; cps, counts per second; Supern., supernatant; dpi, day postinfection.

To investigate the role of RBMX in the regulation of HIV-1 infection, cells with knockdown of RBMX were generated by transducing Jurkat T cells with lentiviruses containing RBMX-specific short hairpin RNAs (shRNAs) (Fig. 1A) and infected with pseudotyped HIV-luc/VSV-G (HIV luciferase/vesicular stomatitis virus G) for 2 days. Then, luciferase activity was measured for viral infection. RBMX knockdown resulted in a 15- to 47-fold enhancement of HIV-1 infection based on the results from five independent experiments (Fig. 1B). When different amounts of HIV-luc/VSV-G were used for infection, RBMX knockdown cells consistently showed a 26- to 44-fold increase in viral infection (Fig. 1C). Conversely, overexpression of RBMX in Jurkat T cells inhibited HIV-1 infection (Fig. 1D and E).

We further confirmed RBMX`s inhibitory role in primary CD4^+^ T cells. Phytohemagglutinin P (PHA-P)-activated CD4^+^ T cells significantly knocked down endogenous RBMX by transducing the cells with lentiviruses containing RBMX-specific shRNA for 3 days (Fig. 1F) and then infecting the cells with replication-competent virus HIV_NL4-3_ for an additional 5 and 7 days. RBMX knockdown increased HIV-1 replication, as demonstrated by increased synthesis of HIV-1 p24^Gag^ and p55^Gag^ proteins as detected by Western blotting (Fig. 1F, left panel), p24^Gag^ capture enzyme-linked immunosorbent assays (ELISAs) (Fig. 1F, right panel), and increased production of infectious viruses in the cell cultural supernatants as quantified by titration in TZM-bl indicator cells (Fig. 1G).

To ensure the above observation is not limited to selective cell types, we went on to examine whether the inhibitory role of RBMX could also be demonstrated in HEK293T cells. Again, the endogenous RBMX in HEK293T cells was knocked down by either transducing the cells with lentiviruses containing RBMX-specific shRNAs (see Fig. S1A in the supplemental material) or transfecting the cells directly with specific small interfering RNAs (siRNAs) (Fig. S2A), and then the cells were infected with HIV-luc/VSV-G for an additional 2 days. Viral infection was monitored by either tracking luciferase activity (Fig. S1B and Fig. S2B) or capture ELISA for quantifying p24^Gag^ production (Fig. S1C), or quantification of HIV-1 *gag* mRNA by real-time PCR (RT-PCR) (Fig. S2C). RBMX knockdown significantly promoted HIV-1 infection and production (Fig. S1B and S1C and Fig. S2B and S2C). Conversely, RBMX overexpression in HEK293T cells inhibited HIV-1 production (Fig. S1D, S1E, and S1F). Taken together, these data demonstrate the modulative role of RBMX on HIV-1 infection.

10.1128/mBio.03424-19.1 FIG S1 RBMX inhibits HIV-1 infection of HEK293T cells. (A to C) RBMX knockdown promotes viral infection. HEK293T cells were infected with lentiviruses containing specific shRNAs to generate cells in which RBMX had been stably knocked down. RBMX expression was detected by Western blotting (A), and cells were infected with HIV-luc/VSV-G (2 ng p24^Gag^) for 48 h, and viral replication was detected by measuring luciferase activity (B) or by quantifying viral production in supernatant with p24^Gag^ capture ELISA (C). (D to F) RBMX overexpression inhibits HIV-1 infection. HEK293T cells were transfected with RBMX-expressing plasmids for 24 h and then infected with HIV-luc/VSV-G for another 48 h. RBMX expression was detected by Western blotting (D), and HIV-1 infection was detected by measuring luciferase activity (E) and p24^Gag^ capture ELISA (F). Data are presented as means ± SD. The results are from one representative experiment from at least three independent experiments. **, *P* < 0.01; ***, *P* < 0.001. Download FIG S1, TIF file, 0.3 MB. Copyright © 2020 Ma et al. 2020 Ma et al. This content is distributed under the terms of the Creative Commons Attribution 4.0 International license.

10.1128/mBio.03424-19.2 FIG S2 RBMX knockdown promotes HIV-1 transcription. HEK293T cells were transfected with RBMX-specific siRNAs for 24 h and then infected with HIV-luc/VSV-G (2 ng p24^Gag^) for an additional 24 h. (A) RBMX expression was detected by Western blotting, (B) HIV-1 infection was monitored by detecting luciferase activity. (C and D) HIV-1 *gag* DNA and the transcribed *gag* mRNA were quantified with real-time PCR (RT-PCR). The *GAPDH* gene was used for normalization. (E) Transcribed viral mRNAs were isolated, and specific primers were used to quantify the initiation and elongation of HIV-1 transcription. Data are presented as means ± SD. The results from one representative experiement from at least three independent experiments are shown. *, *P* < 0.05; **, *P* < 0.01. Abbreviations for elongated viral mRNA transcripts: Pro, proximal; Int, intermediate; Dis, distal. Download FIG S2, TIF file, 0.3 MB. Copyright © 2020 Ma et al. 2020 Ma et al. This content is distributed under the terms of the Creative Commons Attribution 4.0 International license.

### RBMX inhibits the elongation of HIV-1 transcription.

**FIG 2:**
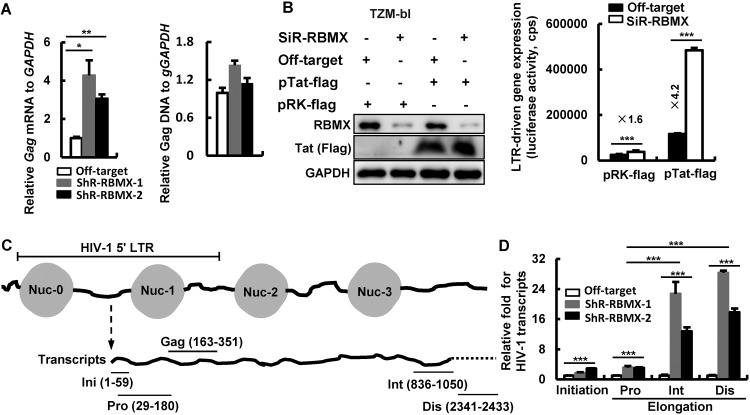
RBMX inhibits HIV-1 LTR-driven transcription. (A) RBMX modulates HIV-1 transcription. Jurkat T cells with or without RBMX knockdown were infected with HIV-luc/VSV-G (2 ng p24^Gag^) for 24 h, and then the HIV-1 proviral *gag* DNA and the transcribed *gag* mRNA were quantified with real-time PCR (RT-PCR). The *GAPDH* gene was used for normalization. (B) RBMX modulates both basal and Tat-driven HIV-1 transcription. TZM-bl cells were cotransfected with RBMX siRNAs, and/or HIV-1-*tat* expressing plasmid pRK-Flag/tat, and/or siRNA off-target control and plasmid vector control. The expression of RBMX and Tat was detected by Western blotting, and HIV-1 5′-LTR-driven gene expression was detected by measuring luciferase activity. (C and D) RBMX modulates the elongation of LTR-driven transcription. Jurkat T cells with or without RBMX knockdown were infected with HIV-luc/VSV-G (2 ng p24^Gag^) for 24 h, transcribed viral mRNAs were isolated, and specific primers were used to quantify the initiation and elongation of HIV-1 transcription. Data are presented as means plus SD. The results from one representative experiment from at least three independent experiments are shown. Values that are significantly different are indicated by bars and asterisks as follows: *, *P* < 0.05; **, *P* < 0.01; ***, *P* < 0.001. Abbreviations for elongated viral mRNA transcripts: Pro, proximal; Int, intermediate; Dis, distal.

RBMX is mainly located in the nucleus, and it is responsible for regulating gene transcription and pre-mRNA splicing (19). Next, we investigated whether RBMX can inhibit HIV-1 transcription. Jurkat cells with or without stable knockdown of RBMX were infected with HIV-luc/VSV-G for 24 h and then quantified for the production of HIV-1 *gag* DNA and mRNA by RT-PCR. RBMX knockdown did not significantly alter HIV-1 *gag* DNA level (Fig. 2A, right panel), but it promoted HIV-1 *gag* mRNA production (Fig. 2A, left panel). Similar results were obtained by using HEK293T cells with knockdown of RBMX, in which there was enhancement of HIV-1 infection and *gag* mRNA production but no change in *gag* DNA level (Fig. S2C and S2D). The above data indicate that RBMX specifically inhibits the HIV-1 transcription step.

To further dissect the molecular details on how RBMX inhibits HIV-1 LTR-driven transcription, TZM-bl cells that have been engineered to contain integrated HIV-1 LTR were cotransfected with specific siRNAs to knockdown RBMX and an HIV-1 *Tat-*expressing plasmid to promote transcription (Fig. 2B, left panel). The results showed that RBMX knockdown promoted both basal and Tat *trans*-activated LTR-driven transcription as measured by luciferase activity (Fig. 2B, right panel).

We then went to gauge the initiation and elongation of HIV-1 LTR-driven transcription by monitoring RT-PCR products with specific primers (Fig. 2C), using previously described methods (29, 30). RBMX knockdown slightly increased the production of initiated viral messenger RNAs (vmRNAs) (1.7- to 2.9-fold) and the elongated vmRNAs proximal (Pro) transcripts (3-fold), whereas the intermediate (Int) and distal (Dis) transcripts showed a 12- to 28-fold increase (Fig. 2D). Similar results have been obtained in HIV-luc/VSV-G-infected HEK293T cells in which RBMX knockdown by siRNA promoted the production of the elongated Int and Dis transcripts of vmRNAs (Fig. S2E). Taken together, these data demonstrate that RBMX mainly impedes the elongation of HIV-1 5′-LTR-driven transcription.

### RBMX interacts with proteins located at the downstream region of HIV-1 5′-LTR.

**FIG 3:**
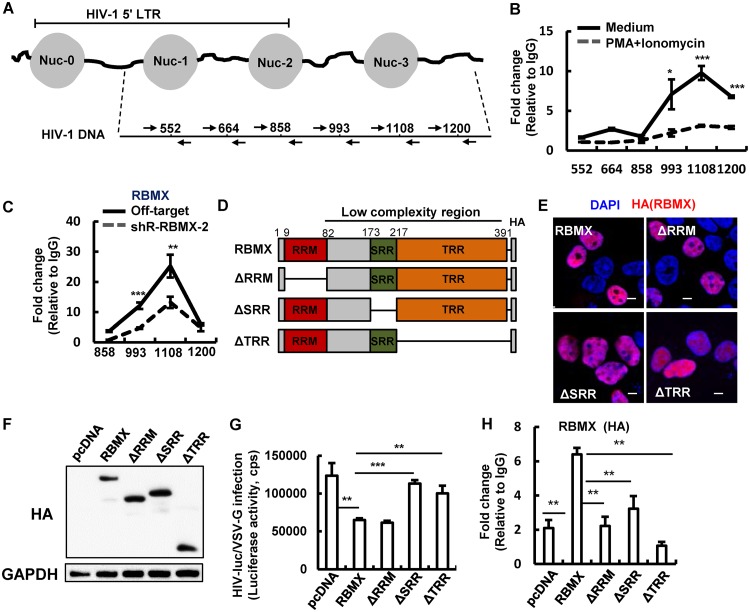
RBMX binds to the HIV-1 5′-LTR downstream region. (A) A schematic diagram of specific primers used for mapping RBMX-binding sites on HIV-1 proviral DNA. (B and C) RBMX binds with HIV-1 proviral DNA. HEK293T cells were infected with HIV-luc/VSV-G (2 ng p24^Gag^) for 48 h, then treated with PMA (10 nM)/ionomycin (1 μM) or without PMA/ionomycin for additional 4 h, and cells were subjected to RBMX ChIP assay. (B) Fragments of HIV-1 LTR and downstream regions covering nucleotides 552 to 1200 were amplified by PCR (B). (C) Jurkat T cells with or without RBMX knockdown were infected with HIV-luc/VSV-G (2 ng p24^Gag^) for 24 h, and RBMX binding with HIV-1 proviral DNA was mapped by ChIP-PCR. (D) A schematic diagram showing wild-type RBMX protein and mutants. A HA tag was added to the C termini of constructs. (E) Cellular locationd of RBMX and mutants. HEK293T cells were seeded on poly-l-lysine-coated microscope slides and transfected with indicated plasmids. Anti-HA antibody was used for immunostaining to indicate RBMX (red), and the nucleus was indicated with DAPI staining (blue). The stained cells were observed under confocal microscopy. Bars, 10 μm. (F to H) Binding of RBMX or mutants with HIV-1 proviral DNA and the inhibition on HIV-1 infection. HEK293T cells were transfected with pCDNA3.1-HA/RBMX or mutant plasmids for 24 h and then infected with HIV-luc/VSV-G (2 ng p24^Gag^) for an additional 48 h. The expression of RBMX or mutants was measured by Western blotting (F), the inhibition on HIV-1 infection mediated by RBMX or mutants was measured by detecting luciferase activity (G), and RBMX or mutants binding the region around the 1108 nucleic acid site of HIV-1 proviral DNA was investigated by ChIP-PCR (H). Data are presented as means plus SD. The results of one representative experiment from three independent experiments are shown. *, *P* < 0.05; **, *P* < 0.01; ***, *P* < 0.001.

RBMX is known to regulate gene expression through an association with promoters (25). To explore whether RBMX can bind to the HIV-1 promoter within the LTR region to modulate HIV gene expression, we performed chromatin immunoprecipitation (ChIP)-PCR assay in HIV-luc/VSV-G-infected HEK293T cells and analyzed binding by using specific primers (Fig. 3A). The mapping results revealed that RBMX did not bind directly to nucleosome 0 (Nuc-0), Nuc-1, or Nuc-2, instead, it was enriched on the HIV-1 5′-LTR downstream region, particularly the 1108 nucleic acid site (Fig. 3B). The treatment with a phorbol-12-myristate-13-acetate (PMA)/ionomycin cocktail to activate LTR significantly reduced RBMX′s binding to this region (Fig. 3B). Similar experiments in Jurkat T cells confirmed the enrichment of RBMX to the region downstream of HIV-1 5′-LTR, especially at the 1108 nucleic acid site, and RBMX knockdown markedly reduced RBMX’s association with this site (Fig. 3C). These data confirmed a specific association between RBMX and HIV-1 5′-LTR downstream region.

RBMX is comprised of three domains: an amino-terminal RNA recognition motif (RRM) that assists RNA splicing, a carboxyl-terminal serine and arginine-rich region (SRR) that serves as enzymatic sites, and a tyrosine-rich region (TRR) that mediates binding with proteins (19, 31). Next, we mapped which of these domains mediated the inhibitory role of RBMX and investigated whether there was direct binding between RBMX and HIV-1 proviral DNA. Truncated RBMX mutants were constructed (Fig. 3D) and used to transfect HEK293T cells for 48 h, with plasmid expressing intact RBMX used as a positive control. A confocal microscopy assay revealed the nucleoplasm location of RBMX, similar to previously described (19); the mutants with the RRM motif deletion (ΔRRM) or the SRR motif deletion (ΔSRR) were also located in nucleoplasm, whereas deletion of the TRR motif (ΔTRR mutant) altered its location to the nucleolus (Fig. 3E). The transfected HEK293T cells were further infected with HIV-luc/VSV-G for an additional 48 h, viral infection was detected, and the ChIP-PCR assay was performed. The ΔRRM mutant was capable of inhibiting HIV-1 infection (Fig. 3F and G) but unable to bind HIV-1 proviral DNA at the 1108 nucleic acid site (Fig. 3H), indicating that direct binding of RBMX with HIV-1 proviral DNA is not responsible for mediating the inhibitory role. Deletion of the SRR motif did not alter RBMX’s cellular location, but it abolished its viral suppressing activity (Fig. 3G), suggesting that SRR motif-mediated interaction with other host protein(s) may be responsible for mediating HIV-1 suppression. These data demonstrate that a direct binding of RBMX with HIV-1 proviral DNA is not required for mediating the inhibition of HIV-1 infection and that interaction between RBMX with other proteins is involved.

### RBMX binds with and maintains H3K9me3 modification to suppress HIV-1 infection.

**FIG 4:**
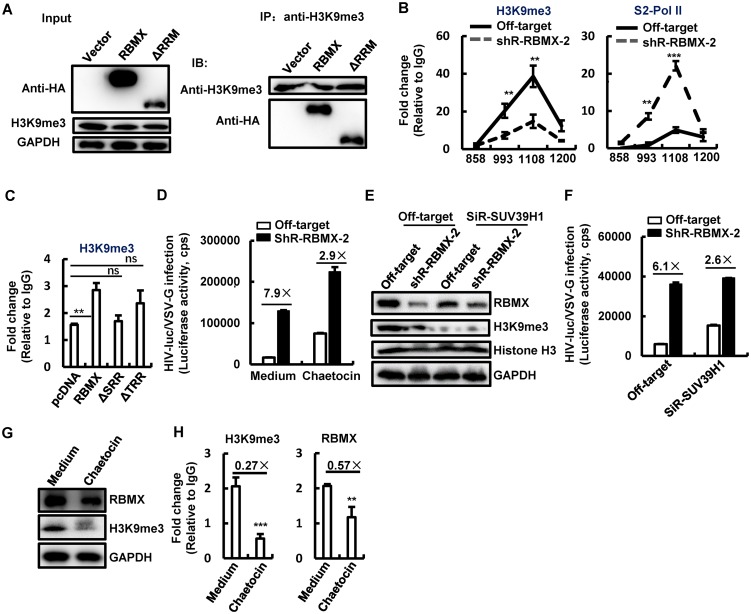
RBMX binds with and maintains H3K9me3 modification for suppressing HIV-1 infection. (A) Association between RBMX and H3K9me3. HEK293T cells were transfected with protein-expressing plasmid pCDNA3.1-HA/RBMX or mutant plasmid ΔRRM. Cell lysates were incubated with H3K9me3-specific antibodies for immunoprecipitation (IP), and the anti-HA tag antibodies were used for immunoblotting (IB). (B) RBMX maintains H3K9me3 modification and blocks the recruitment of pSer2-RNA pol II. Jurkat T cells with or without RBMX stable knockdown were infected with HIV-luc/VSV-G (2 ng p24^Gag^) for 24 h, and cells were harvested and subjected to a ChIP assay for H3K9me3 and pSer2-RNA pol II. (C) H3K9me3 modification maintained by RBMX or mutants. HEK293T cells transfected with pCDNA3.1-HA/RBMX or mutant plasmids and infected with HIV-luc/VSV-G (2 ng p24^Gag^) were subjected to H3K9me3 ChIP-PCR assay. Fragments of HIV-1 proviral DNA around the 1108 nucleic acid site were amplified. (D) Treatment with H3K9me3 inhibitor compromises RBMX-mediated HIV-1 inhibition. Jurkat T cells with or without RBMX stable knockdown were infected with HIV-luc/VSV-G (2 ng p24^Gag^) for 48 h in the presence or absence of chaetocin (50 nM), and HIV-1 infection was monitored by measuring luciferase activity. (E and F) Knockdown of SUV39H1 compromises RBMX-mediated HIV-1 inhibition. Jurkat T cells with or without RBMX stable knockdown were transfected with siRNAs specific for SUV39H1 by electroporation for 24 h and then infected with HIV-luc/VSV-G (2 ng p24^Gag^) for an additional 48 h. H3K9me3 and RBMX expression was detected by Western blotting (E), and HIV-1 infection was monitored by measuring luciferase activity (F). (G and H) Reducing H3K9me3 modification deceases RBMX’s association with HIV-1 proviral DNA. Jurkat T cells were infected with HIV-luc/VSV-G (2 ng p24^Gag^) for 48 h in the presence or absence of chaetocin (50 nM). H3K9me3 expression was detected by Western blotting (G), and the associations of H3K9me3 and RBMX with the HIV-1 5′-LTR downstream region around the 1108 nucleic acid site were investigated by ChIP-PCR. Data are presented as means ± SD. The results from one representative experiment from at least three independent experiments are shown. **, *P* < 0.01; ***, *P* < 0.001; ns, not significant.

RBMX is known to maintain the H3K9me3 modification in order to modulate gene transcription (24). Having demonstrated above that the interaction between RBMX with other proteins is involved for its inhibitory role, we first investigated the association of RXBM with H3K9me3 by immunoprecipitation assay. HEK293T cells were transfected with protein-expressing plasmid pCDNA3.1-HA/RBMX or mutant plasmid ΔRRM, and both proteins were active in inhibiting HIV-1 as demonstrated above. Cell lysates were incubated with H3K9me3-specific antibodies for immunoprecipitation, and the anti-hemagglutinin tag (anti-HA-tag) antibodies were used for immunoblotting. Results showed the association of both wild-type RBMX and ΔRRM mutant with H3K9me3 (Fig. 4A). Next, to examine whether the maintenance of H3K9me3 modification confers RBMX`s inhibitory role for HIV gene expression, we performed ChIP-PCR assay using the HIV-luc/VSV-G-infected Jurkat T cells and analyzed H3K9me3 modification around the 1108 nucleic acid site of HIV-1 proviral DNA, where RBMX binds. Results confirmed the presence of H3K9me3 modification around the 1108 nucleic acid site of HIV-1 proviral DNA (Fig. 4B, left panel), and RBMX knockdown decreased the modification of H3K9me3 (Fig. 4B, left panel). The H3K9me3 modification can maintain the heterochromatin and block the recruitment of positive transcription factors (32). To investigate whether this happens, we performed ChIP-PCR assay using the HIV-luc/VSV-G-infected Jurkat T cells and analyzed the recruitment of phosphorylated RNA polymerase II (RNA pol II). Results showed that RBMX knockdown significantly promoted the recruitment of phosphorylated RNA pol II at Ser-2 (pSer-2 RNA pol II) to the region around the 1108 nucleic acid site of HIV-1 proviral DNA (Fig. 4B, right panel). We next use the RBMX mutants to verify the function of RBMX at the maintenance of H3K9me3 modification. HEK293T cells were overexpressed with either intact or truncated RBMX, infected by HIV-luc/VSV-G for 48 h, and used for ChIP-PCR assay. Both ΔTRR and ΔSRR mutants that lost the domains responsible for protein binding did not inhibit HIV-1 infection and failed to maintain the H3K9me3 modification around the 1108 nucleic acid site (Fig. 4C).

To further confirm that the modification of H3K9me3 around the 1108 nucleic acid site is involved in RBMX-mediated HIV-1 modulation, a lysine-specific histone methyltransferase inhibitor, chaetocin, which reduces H3K9me3 modification by inhibiting the histone methyltransferase effects of SUV39H1 (33), was used to treat Jurkat T cells 4 h after HIV-luc/VSV-G infection. RBMX knockdown increased HIV infection by 7.9-fold, but chaetocin treatment decreased enhancement to 2.9-fold, a 63% suppression (Fig. 4D). We further knocked down the expression of *SUV39H1* with specific siRNAs in Jurkat T cells in which RBMX had been stably knocked down to investigate its effect on RBMX-mediated HIV-1 suppression (Fig. 4E). The additional knockdown of SUV39H1 further reduced RBMX-mediated enhancement for HIV-1 infection from 6.1-fold to 2.6-fold, representing a 57% suppression (Fig. 4F).

To strengthen the finding that H3K9me3 provides the basis for RBMX association with HIV-1 5′-LTR downstream region, Jurkat T cells infected with HIV-luc/VSV-G were further treated with chaetocin, and the reduced modification of H3K9me3 (Fig. 4G) and decreased H3K9me3 association with the 5′-LTR downstream region around the 1108 nucleic acid site (Fig. 4H, left panel) were observed. As expected, RBMX association with this region was significantly reduced (Fig. 4H, right panel). These data demonstrate that H3K9me3 mediates RBMX association with HIV-1 LTR downstream region.

Taken together, these data demonstrate that H3K9me3 mediates RBMX location at the HIV-1 5′-LTR downstream region to maintain the H3K9me3 modification and suppress HIV-1 transcription.

### RBMX maintains HIV-1 latency by modulating viral reactivation from an integrated proviral DNA.

**FIG 5:**
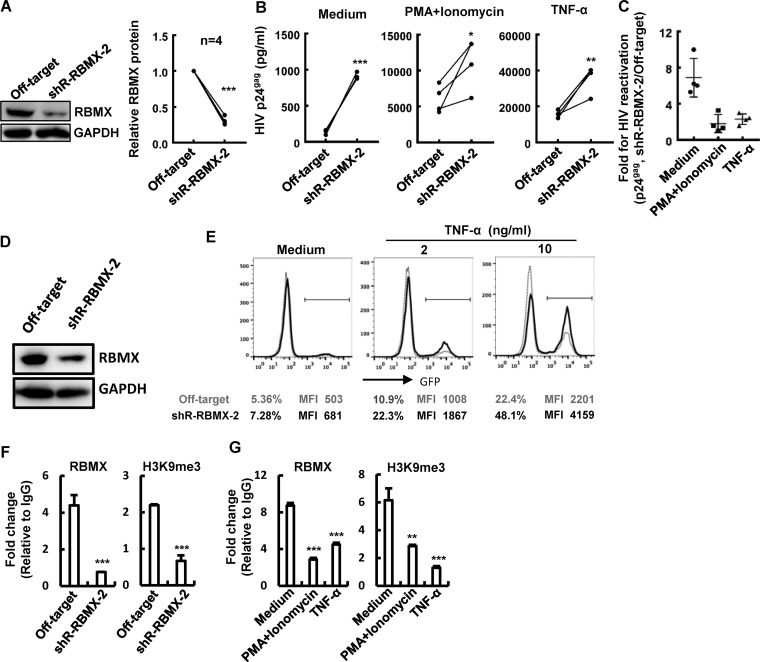
RBMX maintains HIV-1 latency. (A to E) RBMX knockdown promotes HIV-1 reactivation. HIV-1 latently infected ACH2 cells were infected with lentiviruses containing specific shRNAs to generate ACH2 cells in which RBMX was stably knocked down. RBMX expression was detected by Western blotting, and the gel analysis from four cell clones with RBMX stably knocked down were summarized. The parental ACH2 cells and the four cell clones with RBMX stably knocked down were treated with TNF-α (20 ng/ml), PMA (10 nM)/ionomycin (1 μM) for 24 h, cell culture supernatants were collected for monitoring viral production by p24^Gag^ capture ELISA (B), and the fold change for HIV-1 reactivation was calculated (C). The endogenous RBMX in HIV-1 latently infected Jurkat T cells (C11 clone) was knocked down with lentiviruses containing specific shRNAs for 48 h (D), and cells were treated with the indicated concentrations of TNF-α for an additional 24 h. GFP expression indicating viral reactivation was measured by flow cytometry, and the percentage for GFP-positive cells and the mean fluorescence intensity (MFI) were calculated (E). (F and G) RBMX binding with HIV-1 proviral DNA and the maintenance of H3K9me3 modification. ACH2 cells with or without RBMX stably knocked down were treated with TNF-α (20 ng/ml), PMA (10 nM)/ionomycin (1 μM) for 24 h and then were subjected to RBMX and H3K9me3 ChIP assay. Fragments of HIV-1 proviral DNA around the 1108 nucleic acid site were amplified by PCR. Data are presented as means ± SD. *, *P* < 0.05; **, *P* < 0.01; ***, *P* < 0.001.

HIV-1 latency is characterized by the silence of LTR-driven transcription of an integrated proviral DNA (34, 35). The inhibitory role RBMX plays on HIV-1 transcription suggests a potential for it to modulate HIV-1 latency. To investigate this, HIV-1 latently infected ACH2 cells was infected with lentiviruses containing specific shRNAs to knockdown the endogenous RBMX and then used to detect viral reactivation after treatment with tumor necrosis factor alpha (TNF-α) or a mixture of PMA/ionomycin (Fig. 5A). RBMX knockdown significantly promoted HIV-1 reactivation, with a 5- to 11-fold enhancement compared to the medium control group (Fig. 5B and C). To confirm that the observed correlation of RBMX expression and HIV-1 reactivation is not an artifact in a single cell line, we tested this in the HIV-1 latently infected Jurkat T-cell (C11 clone) which harbors an HIV-1 proviral DNA encoding green fluorescent protein (GFP) (13, 36–38). Similar to results obtained in ACH2 cells, the knockdown of endogenous RBMX in C11 cells with shRNAs promoted both basal (medium) and TNF-α-stimulated HIV-1 reactivation (Fig. 5D and E).

The ChIP-PCR assay was also performed in ACH2 cells. RBMX knockdown and treatment with TNF-α or PMA/ionomycin reduced the binding of RBMX to HIV-1 proviral DNA at the 1108 nucleic acid site (Fig. 5F and G, left panels) and concurrently reduced the modification of H3K9me3 at this region (Fig. 5F and G, right panels). Taken together, these results demonstrate that RBMX maintains HIV-1 latency by modulating viral reactivation from an integrated proviral DNA.

## DISCUSSION

Repression of gene transcriptional represents a main mechanism by which HIV-1 latency is maintained (6). Multiply nuclear factors, including hnRNPs, such as hnRNPA2/B1, hnRNP U, have been shown to modulate the transcription of latently infected HIV-1 (39–41). We have previously reported that one hnRNP family member, SAFB1 (scaffold attachment factor B), is capable of inhibiting HIV-1 transcription by preventing the recruitment of RNA pol II to HIV-1 LTR (29). In fact, hnRNPs may function as sensors for viral nucleic acids and induce antiviral responses. For instance, hnRNPA2B1 senses herpes simplex virus 1 DNA to initiate alpha/beta interferon (IFN-α/β) production and enhances STING-dependent cytoplasmic antiviral signaling (42); hnRNP U (SAFA) can sense double-stranded RNA (dsRNA) of both DNA and RNA viruses, and it oligomerizes and activates antiviral responses during viral infections (43). In this study, we report another hnRNP member, RBMX, as a novel viral restriction factor that suppresses HIV-1 infection of CD4^+^ T cells by modulating HIV-1 5′-LTR-driven transcription.

**FIG 6:**
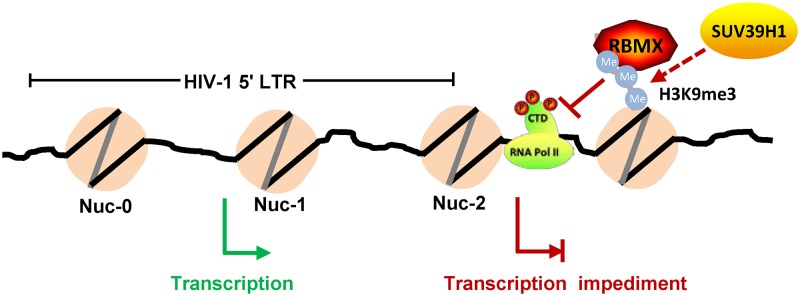
A schematic illustration of RBMX suppressing HIV-1 transcription. RBMX locates at the downstream region around the 1108 nucleic acid site of HIV-1 5′-LTR by associating with H3K9me3; the binding and association maintains the suppressive H3K9me3 modification, leading to a blockage of the recruitment of the positive transcription factor phosphorylated RNA polymerase II (RNA pol II) and the consequential impediment of transcription elongation.

In contrast to a previous report that RBMX binds to the promoter to regulate gene expression (25), we found that RBMX was indirectly enriched on the HIV-1 promoter through association with H3K9me3, and it bound to the LTR downstream region of HIV-1 proviral DNA to maintain the repressive H3K9me3, resulting a blockade of the recruitment of the positive transcription factors and an impediment of transcription elongation (Fig. 6). RBMX has not been reported to have a typical DNA binding motif, the C-terminal SRR and TRR domains are mainly responsible for protein binding (19, 31). However, hnRNPA2B1 was recently reported to use its RRM domain to recognize viral DNA to initiate antiviral signaling (42). In this study, the deletion of RRM in RBMX reduced its binding to HIV-1 proviral DNA but maintained its ability to inhibit HIV-1 infection, indicating that an indirect binding of RBMX with HIV-1 proviral DNA is mediating the inhibitory role. In support of this interpretation, deletion of the SRR motif did not alter the cellular location of RBMX but abolished its HIV-1 suppressing activity, suggesting that SRR motif-mediated interaction with other host protein(s) is required for mediating HIV-1 suppression. Considering the association of RBMX with H3K9me3 in a heterochromatin environment (24, 44) and the fact that the ΔSRR mutant lacking HIV-1 inhibition activity could not maintain the H3K9me3 modification, we infer that the association of RBMX with H3K9me3 is not mediated through a direct binding with HIV-1 proviral DNA.

It is interesting to note that the modification of H3K9me3, even at the location 1 kb away from the transcription start site (TSS), can still inhibit gene transcription (45). H3K9me3 silences HIV-1 transcription, and the removal of this modification by interfering with the expression of related histone methyltransferase or treatment with histone methyltransferase inhibitors can reduce transcription repression and reactivate HIV-1 from latency (8, 10, 46, 47). In keeping with this knowledge, in this study, we found that the knockdown of methyltransferase SUV39H1 or treatment with the histone methyltransferase inhibitor chaetocin compromised RBMX-mediated HIV-1 suppression. H3K9me3 associates with TSS downstream region and impedes RNA pol II-mediated transcription elongation (48–50). In accordance with this, we observe an association of H3K9me3 with the region downstream of HIV-1 LTR to block the recruitment of phosphorylated RNA RNA pol II, thus hindering HIV-1 transcription elongation. We recognize that the epigenetic change was derived by using transformed cells, and validation by using primary T cells would be helpful in a future study.

The modulation of RBMX on HIV-1 5′-LTR-driven transcription confirmed its function in maintaining HIV-1 latency at postintegrational steps. By using the HIV-1 latently infected ACH2 cells and Jurkat T cells, we demonstrate that RBMX modulates HIV-1 reactivation from an integrated proviral DNA and maintains HIV-1 latency. Treatment with TNF-α or latency reverse agent PMA/ionomycin removes RBMX association with HIV-1 proviral DNA and reactivates viral latency. Overall, this study demonstrates that RBMX may be a new host target to which HIV-1 latency and reactivation could be modulated in order to achieve HIV eradication. Multiple mechanisms for explaining the establishment, maintenance, and reversal of HIV-1 latency have been proposed, and several HIV-1 latency models have hence been established (51–55). Thus, multiple disparities remain in these latency modulation models regarding virus-encoded programs such as Tat positive-feedback circuitry, posttranscriptional splicing feedback, and the viral evolution property for optimizing transmission while reducing viral extinction during mucosal infection (52–55), and the T-cell subtype status and cellular signaling have been proposed as well for explaining the modulation of HIV latency (51). These primary models including the resting CD4^+^ T cells isolated from patients under combination antiretroviral therapy should provide more physiologically relevant cellular models to validate our results using cell lines.

Taken together, in this study, we have identified a novel role of RBMX for suppressing HIV-1 transcription and infection. Our findings facilitate a better understanding of host modulation of HIV-1 infection and latency and provide a potential new host target site for the development of HIV-1 therapies.

## MATERIALS AND METHODS

### Ethics statement.

The Medical Ethics Review Committee of the Institut Pasteur of Shanghai, Chinese Academy of Sciences approved the use of human cells.

### Cells.

Buffy coat from healthy donors was purchased from Shanghai Changhai hospital and used to isolate human peripheral blood mononuclear cells (PBMCs) by using Ficoll-Paque density gradient centrifugation. CD4^+^ T cells were separated from PBMCs by using CD4 antibody-coated magnetic beads (Miltenyi Biotec) and maintained in RPMI 1640 medium supplemented with 10% fetal bovine serum (FBS) and penicillin-streptomycin in the presence of 20 IU/ml recombinant, human interleukin 2 (rhIL-2). HeLa cell-derived TZM-bl cell line was a gift from Paul Zhou (Institut Pasteur of Shanghai, Chinese Academy of Sciences, Shanghai, China). The HIV-1 latently infected CD4^+^ CEM cell line ACH2 was provided by Shi-Bo Jiang (Fudan University, Shanghai, China). HIV-1 latently infected Jurkat T cell line (C11 clone) was provided by Huan-Zhang Zhu (Fudan University, Shanghai, China). Human embryonic kidney cells (HEK293T) and TZM-bl cells were cultured in Dulbecco’s modified Eagle medium (DMEM) (HyClone) containing 10% FBS (HyClone), 100 U/ml penicillin, and 100 μg/ml streptomycin. Cells were grown at 37°C under 5% CO_2_.

### Virus stock and infection assay.

Calcium phosphate-mediated transfection of HEK293T cells was used to generate virus stocks. Pseudotyped HIV-luc/VSV-G was obtained by cotransfection with luciferase reporter HIV-1 proviral plasmid pNL-Δenv-luc and the expression plasmid vesicular stomatitis virus G (VSV-G) protein. pNL-Δenv-luc is an env-deleted and nef-inactivated HIV-1 proviral construct as described previously (13, 56, 57). Replication-competent virus HIV_NL4-3_ was obtained by transfecting HIV-1 vectors of the pNL4-3 plasmid. These plasmids were kindly provided by Li Wu (The Ohio State University, USA). Harvested supernatants that contained viral particles were filtered and quantified with p24^Gag^ capture ELISA.

Cells were infected with HIV-luc/VSV-G or replication-competent virus HIV_NL4-3_ for the indicated time, and after washing, cells were further cultured for 2 days (cell line) or 5 to 7 days (primary T cells). Viral infection was detected by measuring the luciferase activity from the cell lysates or detecting viral production in the supernatant by p24^Gag^ capture ELISA. HIV-1 production was also titrated in TZM-bl indicator cells which contain LTR-driven luciferase reporter. Briefly, equal amounts of cell culture supernatant containing replication-competent HIV_NL4-3_ were used to infect TZM-bl cells for 48 h, and HIV-1 infection was detected by measuring the luciferase activity.

### Transfection with plasmids, shRNAs, and siRNAs.

cDNA of RBMX was amplified from HEK293T cells and cloned into pcDNA3.1 with a hemagglutinin (HA) tag at the carboxyl terminus. RBMX mutants were constructed from the pcDNA3.1-HA-RBMX. Two RBMX short hairpin RNAs (shRNAs) and off-target shRNA were cloned into PLKO.1-puro (puro stands for puromycin) vector (Addgene), and the sequences were as follows: off-target shRNA, 5′-TTC TCC GAA CGT GTC ACG TAT-3′; Sh-RBMX-1: 5′-TTT CTT GTC TGC CAA CCC GAT C-3′; Sh-RBMX-2: 5′-TTT TGT TTC TTT GAA CTG GGA T -3′. A mixture of small interfering RNAs (siRNAs) used for RBMX knockdown or SUV39H1 knockdown was synthesized (Ribobio, Guangzhou, China). The sequences were as follows: off-target siRNA, 5′-UUC UUC GAA CGU GUC ACG UTT-3′; si-RBMX-1, 5′-CAA GUU CUC GUG AUA CUA G-3′; si-RBMX-2, 5′-GUG GAA GUC GAG ACA GUU A-3′; si-SUV39H1-1, 5′-GGG TCC GTA TTG AAT GCA AGT-3′; si-SUV39H1-2, 5′-GCA CAA GTT TGC CTA CAA TGA-3′. Plasmids, shRNAs, and siRNAs were transfected into HEK293T cells by Lipofectamine 2000 (Invitrogen) or Jurkat T cells by electroporation (NEPA 21, NEPA GENE).

### Real-time PCR (RT-PCR).

Total cellular RNA from cells was isolated by TRIzol reagent (Invitrogen) and reverse transcribed into cDNA using ReverTra Ace quantitative PCR (qPCR) real-time (RT) Master Mix (Toyobo). Total DNA from cells was obtained with the QIAamp DNA blood minikit (Qiagen). Real-time PCR was performed by using the Thunderbird SYBR qPCR Mix (Toyobo) on the ABI 7900HT real-time PCR system. Primers were listed as below: for *gag*, forward, 5′-GTG TGG AAA ATC TCT AGC AGT GG-3′ and reverse, 5′-CGC TCT CGC ACC CAT CTC-3′; for *GAPDH*, forward, 5′-ATC CCA TCA CCA TCT TCC AGG-3′ and reverse, 5′-CCT TCT CCA TGG TGG TGA AGA C-3′.

### Immunoprecipitation and immunoblotting.

Cells were lysed in radioimmunoprecipitation assay (RIPA) buffer {50 mM HEPES (4-(2-hydroxyethyl)-1-piperazineethanesulfonic acid) (pH 7.4), 150 mM NaCl (sodium chloride), 0.5 mM EGTA [ethylene glycol-bis (β-aminoethyl ether)-*N*,*N*,*N*′,*N*′-tetraacetic acid], 1% protease inhibitor cocktail (Sigma), 1 mM sodium orthovanadate, 1 mM NaF (natrium fluoride), 1% (vol/vol) Triton X-100, and 10% (vol/vol) glycerol} for 1 h on ice with brief vortexing every 10 min. After centrifugation for 10 min at 12,000 × *g*, the lysates were incubated with indicated antibody at 4°C overnight. Dynabeads protein G/A were added into each sample at 4°C for 2 h for immunoprecipitation. The immunoprecipitates were separated by sodium dodecyl sulfate-polyacrylamide gel electrophoresis (SDS-PAGE) and analyzed by immunoblotting. For immunoblotting, cells were lysed for 1 h at 4°C in ice-cold RIPA buffer. After centrifugation for 10 min at 12,000 × *g*, supernatant was boiled in reducing SDS sample loading buffer and analyzed by SDS-PAGE. Specific primary antibodies were used, followed by horseradish peroxidase-conjugated goat anti-mouse IgG or goat anti-rabbit IgG (Sigma) as the secondary antibodies. One percent of total lysates was used as the input.

### Chromatin immunoprecipitation (ChIP).

ChIP assay was performed as previously described (58). Cells were cross-linked with 1% formaldehyde for 10 min at room temperature and quenched with 0.125 M glycine for 5 min. After lysis, chromatin was sheared by use of a sonicator for a total of 12 min (2 s on and 6 s off) on ice to obtain DNA fragments of 200 to 500 bp. Five percent of the total sheared chromatin DNA was used as input sample. Other sheared chromatin was incubated overnight at 4°C with an antibody against pSer-2 RNA pol II (abcam), H3K9me3 (abcam), RBMX (abcam), or rabbit IgG (Cell Signaling), followed by incubating with 40 μl of protein A magnet beads for 4 h. After washing and reversing cross-link, the input and immunoprecipitated DNA was purified and analyzed by real-time PCR, using primers specifically targeting nucleotide positions of HIV-1 proviral DNA, Nuc-1, or nucleotide 552: forward, 5′-TCT CTG GCT AAC TAG GGA ACC-3′, and reverse, 5′-CTA AAA GGG TCT GAG GGA TCT C-3′; nucleotide 664, forward, 5′-GTG TGG AAA ATC TCT AGC AGT G-3′, and reverse, 5′-CTT CAG CAA GCC GAG TCC-3′; Nuc-2 or 858, forward, 5′-AGA GAT GGG TGC GAG AGC-3′, and reverse, 5′-ATT AAC TGC GAA TCG TTC TAG C-3′; 993, forward, 5′-CCT GGC CTT TTA GAG ACA TCA G-3′, and reverse, 5′-GCA CAC AAT AGA GGA CTG CTA T-3′; 1108, forward, GTG TGC ATC AAA GGA TAG ATG T-3′; 1200, forward, 5′-AGC AGC TGA CAC AGG AAA CAA C-3′, and reverse, 5′-CCC ATG CAT TTA AAG TTC TAG G-3′. The primer number represents the middle site of the RT-qPCR product.

### Assays for HIV-1 reactivation.

HIV-1 latently infected ACH2 cells or Jurkat T cells (C11 clone) were infected with lentivirus containing shRNAs specific for RBMX or off-target shRNAs, then stimulated with TNF-α (20 ng/ml for ACH2, 2 and 10 ng/ml for C11) or PMA (10 nM)/ionomycin (1 μM) for 24 h. Viral reactivation in ACH2 was measured by quantifying produced viral particles in the supernatant by p24^Gag^ capture ELISA or by measuring GFP expression in C11 cells.

### Confocal microscopy.

HEK293T cells were cultured on polylysine-coated glass coverslips and transfected with pcDNA-RBMX or truncated pRBMX for 24 h. Cells were then fixed with 4% paraformaldehyde (Sigma-Aldrich), permeabilized with 0.3% Triton X-100 and blocked with 10% goat serum. Specific antibodies for HA tag were incubated with cells overnight at 4^0^C, followed with secondary antibodies of Alexa Fluor 555-labeled IgG (2 mg/ml; Invitrogen). Nuclei were indicated with 4′,6′-diamidino-2-phenylindole (DAPI) (Invitrogen). Slides were mounted with fluorescent mounting medium (Dako) and observed using a laser scanning confocal microscope (Olympus FV-1200).

### Statistical analysis.

Statistical analysis was performed using an unpaired *t* test with SigmaStat 2.0 (Systat Software, San Jose, CA, USA).
