# Supplementary figures and images for: X-Linked RNA-Binding Motif Protein Modulates HIV-1 Infection of CD4+ T Cells by Maintaining the Trimethylation of Histone H3 Lysine 9 at the Downstream Region of the 5′ Long Terminal Repeat of HIV Proviral DNA
Source: mBio. 2020 Apr 21;11(2):e03424-19. doi: 10.1128/mBio.03424-19 (PMC7175097; doi:10.1128/mBio.03424-19)

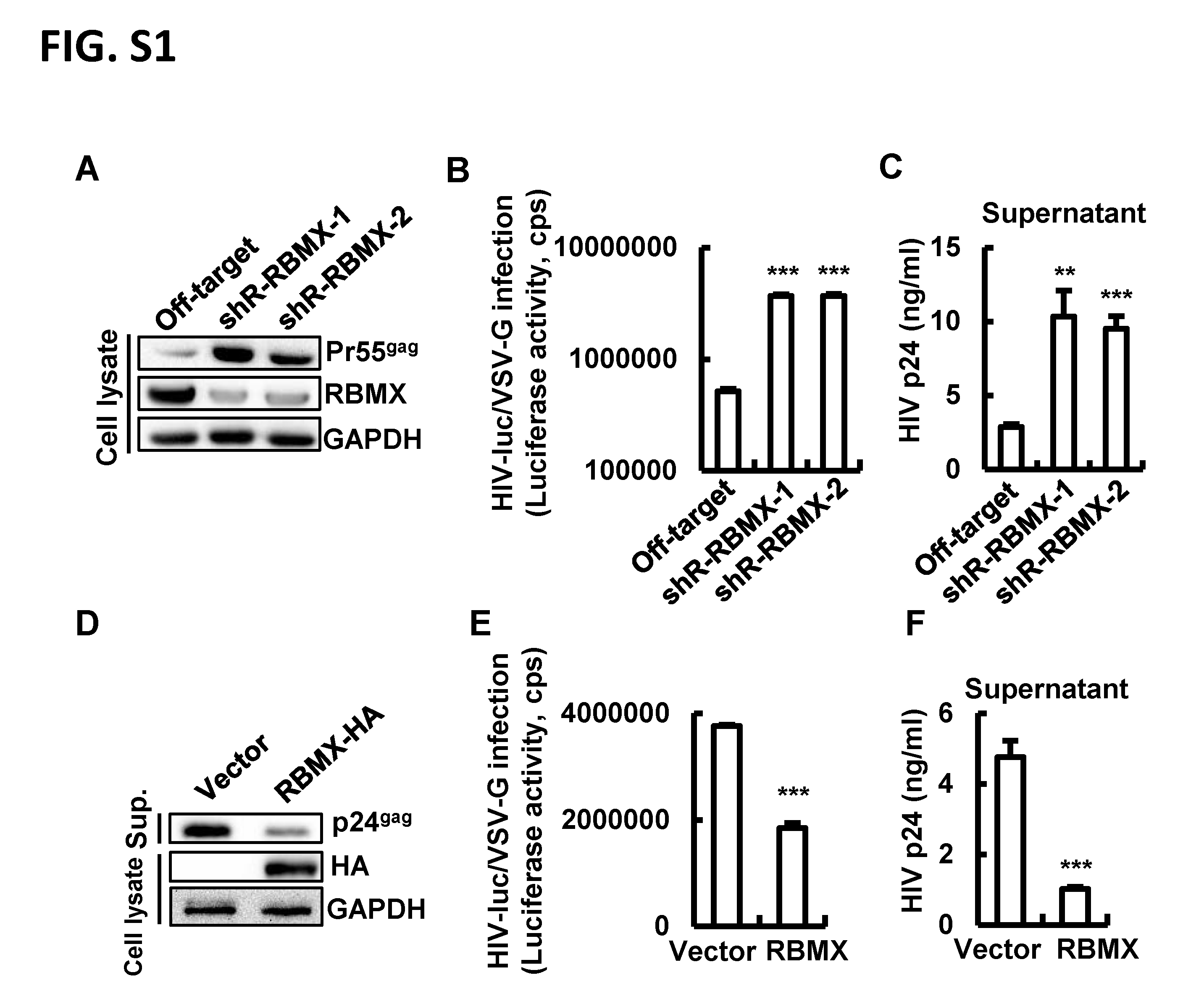

Supplement: FIG S1 [file mBio.03424-19-sf001.tif]

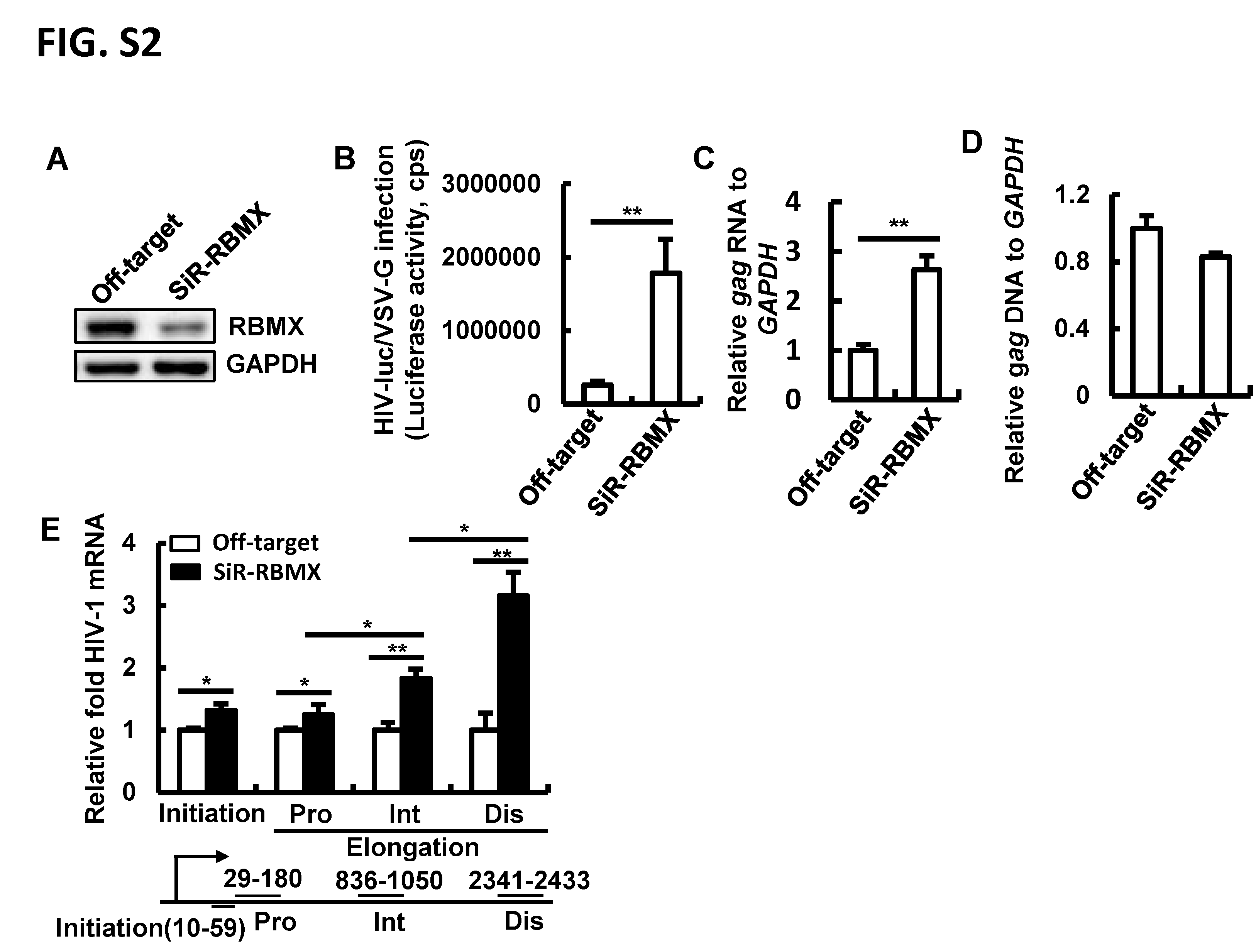

Supplement: FIG S2 [file mBio.03424-19-sf002.tif]
